# Supplementary material for: Identification of photoperiod-induced specific miRNAs in the adrenal glands of Sunite sheep (Ovis aries)
Source: Front Vet Sci. 2022 Jul 22;9:888207. doi: 10.3389/fvets.2022.888207 (PMC9354845; doi:10.3389/fvets.2022.888207)
Supplement: Supplementary file 1 [file Data_Sheet_1.ZIP › Supplementary file/miRDeep2 file.zip!/miRDeep2 file/Novel_Structure/SP42A3/Novel_436.pdf]

Provisional ID	:	NC_019469.2_19176
Score total	:	1.9
Score for star read(s)	:	3.9
Score for read counts	:	-4.9
Score for mfe	:	1.4
Score for randfold	:	1.6
Score for cons. seed	:	
Total read count	:	2
Mature read count	:	1
Loop read count	:	0
Star read count	:	1

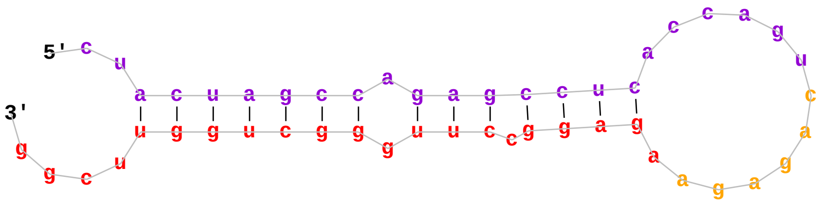

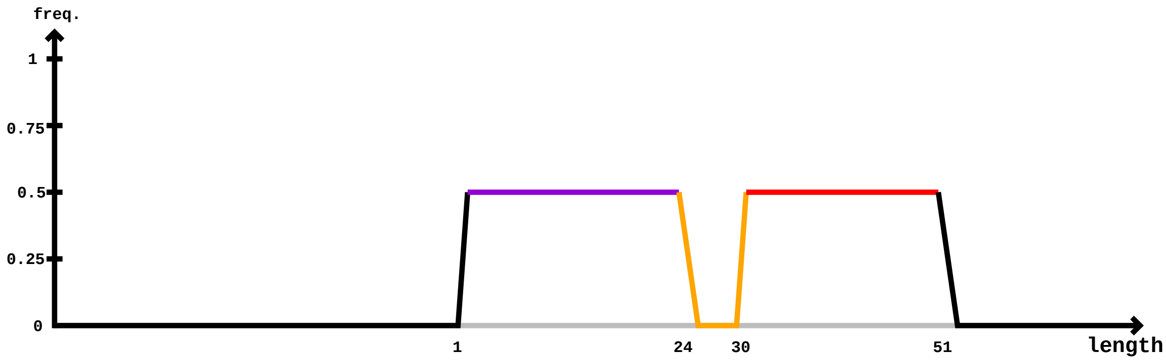


```
                                       Star                       Mature
5'- ggguugcccauaccuuccaugugaccgcucagggacaucuucuacuagccagagccucaccagucagagaagaggccuugggcugguucggaaacuugucccacucuuuaa  -3'   obs
    ggguugcccauaccuuccaugugaccgcucagggacaucuucuacuagccagagccucaccagucagagaagaggccuugggcugguucggaaacuugucccacucuuuaa         exp
    .((((((.............)))))).....((((((..(((((((((((.(((((((.............)))).))).)))))))..))))...)))))).........    reads      mm        sample
    .........................................cuacuagccagagccucaccagu...............................................    1          0         seq
    ......................................................................agaggccuugggcugguucgg....................    1          0         seq
```
